# Transcriptomic Insights Into Alzheimer's Disease: Differentially Expressed Genes and Cholesterol Metabolism

**DOI:** 10.1002/cns.70833

**Published:** 2026-03-19

**Authors:** Rui Sun, Xu Wang, Zaibao Wang, Chunliu Li, Qing Shao, Xiangru Liu, Hongrui Zhu, Sheng Wang, Keqiang He

**Affiliations:** ^1^ Department of Anesthesiology, the First Affiliated Hospital of USTC, Division of Life Sciences and Medicine University of Science and Technology of China Hefei Anhui China; ^2^ Department of Anesthesiology Graduate School of Bengbu Medical University Bengbu Anhui China

**Keywords:** Alzheimer's disease, cholesterol metabolism, machine learning, mendelian randomization

## Abstract

**Background:**

Alzheimer's disease (AD) is a progressive neurodegenerative disorder characterized by cognitive decline and memory impairment, posing significant challenges to affected individuals, their families, and healthcare systems globally. With projections indicating that the prevalence of AD could escalate to 152 million cases by 2050, there is an urgent need to elucidate the underlying mechanisms driving this condition. Additionally, developing effective diagnostic tools to aid in its early detection and management is crucial.

**Methods:**

In this study, we utilized a combination of Mendelian randomization and advanced machine learning techniques to analyze transcriptomic data from five distinct cohorts of Alzheimer's Disease (AD) patients. After addressing batch effects, we identified differentially expressed genes (DEGs) between the AD and control groups. Mendelian randomization analysis was conducted to assess the causal relationships between DEGs and AD risk. A Venn diagram was subsequently used to identify genes associated with cholesterol metabolism from the screened gene set. The shared DEGs were subjected to functional enrichment analyses. Furthermore, immune analysis was quantified using Gene Set Enrichment Analysis (GSEA). A diagnostic model for AD was developed by evaluating 113 combinations of 12 machine learning algorithms with 10‐fold cross‐validation on the training datasets, followed by external validation on test datasets. Finally, immunofluorescence staining was performed on mouse brain slices to verify the expression level of KLHL21.

**Results:**

Our analyses identified a substantial number of differentially expressed genes (DEGs) demonstrating significant differences between Alzheimer's disease (AD) patients and control groups. Among these, we identified 29 genes associated with AD, with 21 of them linked to cholesterol metabolism, highlighting its pivotal role in the disease's pathogenesis. From this set, we developed a robust 8‐gene diagnostic signature (comprising CHSY1, FIBP, DHCR24, HVCN1, KIFAP3, KLHL21, LETMD1, and SLC25A29), which outperformed existing AD diagnostic models in both training and testing cohorts. Additionally, complementary animal experiments were conducted to validate the biological relevance of these genes, further elucidating their roles in AD pathology.

**Conclusions:**

Our research identified critical genes and proposed novel pathways for early diagnosis and potential therapeutic interventions, paving the way for enhanced clinical applications in Alzheimer's disease management.

## Graphical abstract

Our analysis revealed numerous differentially expressed genes between Alzheimer's disease (AD) patients and controls, identifying 29 ad‐related genes, with 21 linked to cholesterol metabolism, underscoring its key role in AD pathogenesis. We developed a robust 8‐gene diagnostic signature that surpassed existing AD diagnostic models in both training and testing cohorts. Animal experiments confirmed the biological relevance of these genes, further clarifying their roles in AD pathology.

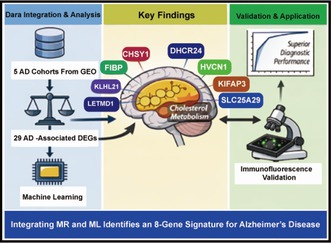

## 1 Introduction

Alzheimer's disease (AD) is a common neurodegenerative disorder marked by progressive cognitive decline and memory loss [1]. It significantly impacts patients' quality of life and places substantial economic burdens on families and healthcare systems worldwide [2]. With an aging population, the incidence of AD is projected to increase significantly, potentially affecting 152 million people by 2050 [3]. Current therapeutic approaches, encompassing both pharmacological interventions and non‐pharmacological strategies, have demonstrated limited efficacy in symptom alleviation or in decelerating disease progression, thereby highlighting the pressing need for a comprehensive understanding of the underlying pathophysiological mechanisms and discovering novel biomarkers for early diagnosis and intervention [4, 5]. Extensive research has elucidated complex interactions among genetic, environmental, and lifestyle factors contributing to AD onset and progression [6, 7, 8, 9, 10]. Specifically, genetic studies have identified numerous risk alleles associated with AD, with particular emphasis on the apolipoprotein E (ApoE) gene, which is integral to lipid metabolism and has been implicated in the pathogenesis of AD [11]. Furthermore, transcriptomic analyses have illuminated alterations in gene expression associated with the disease, revealing potential biomarkers that may enhance early detection and inform therapeutic strategies [12]. However, researchers still face considerable gaps in understanding how these genetic elements affect the expression of key genes in AD and influence clinical outcomes.

Given these challenges, this study aims to address these gaps by applying advanced methodologies such as transcriptomic analysis, Mendelian randomization, and machine learning. We focus on identifying target genes causally related to AD by analyzing differentially expressed genes (DEGs) in AD alongside genes involved in cholesterol metabolism, a pathway increasingly recognized for its role in the disease's etiology [13]. By integrating these methods, we can thoroughly evaluate the relationships among genetic variations, gene expression patterns, and the clinical features of AD. This integrated approach helps discover new therapeutic targets and diagnostic markers.

The research method involves a multi‐layered analysis approach. Initially, it focuses on identifying DEGs through a comprehensive analysis of transcriptome data. Following this, causal relationships between these identified genes and AD are then established using Mendelian randomization techniques. Next, the analysis focuses on identifying genes associated with cholesterol metabolism among the DEGs. Finally, predictive models for early diagnosis are developed by employing machine learning algorithms. By combining these diverse methodologies, this study seeks to elucidate the complex interactions between genetic predisposition, gene expression, and cholesterol metabolism in the context of AD [14]. This approach ultimately contributes to the development of more effective diagnostic and therapeutic strategies.

In conclusion, exploring the relationship between DEGs and cholesterol metabolism in AD provides important findings that deepen our understanding of the disease's fundamental mechanisms. This research aims to offer valuable insights that may guide future therapeutic interventions and enhance patient outcomes, ultimately tackling the increasing public health challenge presented by Alzheimer's disease.

## 2 Materials and Methods

### 2.1 Data Collection

The datasets GSE138260, GSE293878, GSE36980, GSE37263, and GSE5281, containing microarray data on gene expression, were collected from the Gene Expression Omnibus (GEO) database (https://www.ncbi.nlm.nih.gov/geo/). Summary‐level statistical data for AD were obtained from the R10 release of the FinnGen GWAS results (https://r10.finngen.fi/), and single‐nucleotide polymorphisms (SNPs) of interest were sourced from the NHGRI‐EBI GWAS Catalog (https://www.ebi.ac.uk/gwas). Lead SNPs that achieved genome‐wide significance (*p* < 5 × 10^−8^) were identified within the specified genomic loci. In instances where multiple independent signals were detected, all significant SNPs with a linkage disequilibrium threshold of r^2^ < 0.2 were included for further analysis. A gene list related to cholesterol metabolism was retrieved from the GeneCards database (https://www.genecards.org/).

### 2.2 Batch Effect Removal

The five Alzheimer's disease datasets (GSE138260, GSE293878, GSE36980, GSE37263, and GSE5281) were combined before differential analysis. To correct for batch effects, the ‘ComBat’ function from the ‘sva’ package (version 3.5.0) was applied [15]. Principal component analysis (PCA) was used to compare data quality before and after batch effect removal to evaluate the correction's efficacy.

### 2.3 Identification of Differentially Expressed Genes (DEGs)

The ‘Limma’ package in R software [16] was employed to determine DEGs between Alzheimer's disease and non‐demented control datasets, with criteria set at |log2 FC| > 0.585 and a *P*‐value < 0.05. The results were visualized through volcano and heatmap plots using R software (version 4.2.2) with the ‘ggplot2’ and ‘pheatmap’ packages.

### 2.4 Functional Enrichment for Hub Genes

The ‘enrich’ function from the relevant R package was employed to perform enrichment analysis of hypoxia‐associated genes, focusing on KEGG pathways, Gene Ontology (GO), and Disease Ontology (DO) terms.

### 2.5 Mendelian Randomization Analysis

eQTL data were obtained from the FinnGen eQTL database (https://r10.finngen.fi/). A Mendelian randomization (MR) analysis was carried out to determine the causal association between differentially expressed genes (DEGs) and Alzheimer's disease risk, employing summary statistics from the genome‐wide association study (GWAS). In this MR analysis, DEGs acted as the exposure variable, while Alzheimer's disease was the outcome variable. Instrumental variables were chosen as single‐nucleotide polymorphisms (SNPs) that were strongly associated with the exposure but not directly associated with confounders or the outcome except through the exposure. Then, SNPs for the MR analysis were selected based on a strong correlation (*P*‐value < 5 × 10^‐8) and absence of linkage disequilibrium (r^2^ < 0.001). Five different MR analysis techniques, including inverse variance weighting (IVW), weighted median, weighted mode, simple mode, and MR‐Egger regression, were employed to statistically analyze the robustness of the MR results. Because IVW has higher statistical power for effect estimation, it was utilized as the primary method for the MR analysis instead of the other methods. The aforementioned statistical analyses were conducted using R software (version 4.2.1; R Foundation for Statistical Computing) with the TwoSampleMR package (version 0.6.6). The results were visualized using the ggplot2 package. Statistical significance was determined at a *P*‐value of less than 0.05, and the findings were presented as odds ratios (OR) with 95% confidence intervals (CIs). We conducted the following sensitivity analyses to assess key MR assumptions. First, we calculated the F‐statistic for each IV to evaluate potential weak instrument bias, with an average F‐statistic of 17.63, confirming that the majority of IVs exceeded the recommended threshold of F > 10. Second, to examine the presence of horizontal pleiotropy, we performed MR‐Egger intercept tests, and all results were non‐significant (*p* > 0.05). Third, heterogeneity among the IVs was assessed using Cochran's Q statistic, and no significant heterogeneity was detected. Finally, leave‐one‐out sensitivity analysis was conducted to determine if any single SNP disproportionately influenced the overall MR estimates.

### 2.6 Machine Learning for Constructing Diagnostic Models

Following the MR analysis, machine learning approaches were employed to construct diagnostic models. A total of 113 algorithm combinations were generated from 12 machine learning algorithms to identify consistent regulatory genes with strong predictive accuracy and stability. Random forest (RF), Lasso, Ridge, elastic net (Enet), stepwise generalized linear model (stepGLM), support vector machine (SVM), glmBoost, linear discriminant analysis (LDA), and gradient boosting machine (GB) were among the algorithms used. GSE5281 has the largest sample size, and it was included as the training dataset; the other 4 datasets were applied for validation work. To excavate more accurate, stable, and consistent signatures, we integrated up to 10 various machine learning methods. Some of them can perform feature selection, such as Lasso, stepwise Cox, CoxBoost, and RSF. As a result, we combined these algorithms to find a new consensus signature. Based on the leave‐one‐out cross‐validation (LOOCV) framework, 113 algorithm combinations were combined to fit the prediction model. The algorithm combination with the highest average AUC value was considered the optimal model.

### 2.7 Analysis of Protein Interaction Networks and Plotting of ROC Curves

Using the STRING database, the protein interaction network of hub genes was constructed and visualized through Cytoscape. The ‘pROC’ R package was used to perform Receiver Operating Characteristic (ROC) analysis on the hub genes.

### 2.8 Gene Set Enrichment Analysis (GSEA)

Enrichment analysis of all genes was carried out using the GSEA software (http://software.broadinstitute.org/gsea/index.jsp). The analysis was conducted on a pre‐ranked gene list, where genes were ranked based on the correlation between their expression and the KLHL21 expression level (or based on log2 fold change from differential expression analysis, as applicable). The Hallmark (H) gene set collection from the Molecular Signatures Database (MSigDB) was used as the reference. Default parameters were employed with 1000 gene set permutations. Gene sets with a false discovery rate (FDR) q‐value of less than 0.25 were considered statistically significant, as recommended by the GSEA developers. Nominal *p*‐values are also reported in the full results. The complete GSEA output for all significant hallmark pathways is provided in Table S1. For a visual summary in the main figures, the top 10 most significantly enriched pathways, ranked by their normalized enrichment score (NES), are displayed.

### 2.9 Analysis of the Immune Landscape and Genetic Correlations

The CIBERSORT algorithm estimated the makeup of immune cells infiltrating each tumor sample and evaluated the differences in immune cells between normal and diseased groups. Immune cell profiles related to hub genes were then assessed based on the infiltration results. The limma package was used to analyze correlations among hub genes.

### 2.10 Animal Studies

APP23 mice were obtained from Professor Feng Gao (University of Science and Technology of China, Hefei, China). Mouse brains were harvested and preserved in 4% paraformaldehyde at 4°C overnight. They were then transferred to 30% sucrose at 4°C until they settled at the bottom of the tube. The brains were sliced into 40‐μm sections using a cryostat. The sections were washed three times with PBS for 10 min each, permeabilized with 0.25% Triton X‐100 for 20 min, and blocked with a buffer containing 0.1% Triton X‐100 and 3% normal goat serum diluted in PBS for 1 h. The sections were then incubated with primary antibodies. On the subsequent day, after being washed five times with PBS for 10 min each, the brain slices were incubated with fluorescent secondary antibodies for one hour in the dark and then washed five times again with PBS for 10 min each. Secondary antibodies were obtained from CST and diluted 1:500. DAPI staining was performed during the washing steps. Finally, the brain slices were mounted using the appropriate mounting medium and left to dry. Fluorescent images were acquired using a Leica DM6B fluorescent microscope and ZEN software. Fluorescent images were processed and analyzed with Adobe Photoshop CS and ImageJ. Adjustments to brightness and contrast were applied consistently to all images in the set. The normalized intensity values were obtained by dividing the integrated intensity values by the area of the image positively stained with DAPI.

### 2.11 qPCR and Western Blot Analysis

Total RNA was extracted from hippocampal tissues using RNAiso Plus (TaKaRa). One microgram was converted to cDNA with a PrimeScript RT kit (TaKaRa). qPCR was performed with SYBR master mix on an ABI StepOne system, using Gapdh as a reference and 40 cycles of 95°C for 20 s, 95°C for 30 s, and 60°C for 30 s. The 2 − ΔΔCq method analyzed the results. Primer sequences: KLHL21 forward 5′‐CTGGTTACGGTCGACTGCTACA‐3′, reverse 5′‐TCTCCACACGCAGTCATAGAGC‐3′; GAPDH forward 5′‐CATCACTGCCACCCAGAAGACTG‐3′, reverse 5′‐ATGCCAGTGAGCTTCCCGTTCAG‐3′.

The hippocampal tissues were subjected to lysis using radioimmunoprecipitation assay (RIPA) buffer supplemented with protease inhibitors (Beyotime Biotechnology). Protein concentrations were quantified employing a bicinchoninic acid (BCA) protein assay kit (Sangon Biotech). Subsequently, 50 μg of total protein was resolved via 10% sodium dodecyl sulfate‐polyacrylamide gel electrophoresis (SDS‐PAGE) and transferred onto polyvinylidene difluoride (PVDF) membranes. Primary antibodies targeting GAPDH and AHR (both procured from Proteintech Group), along with appropriate horseradish peroxidase (HRP)‐conjugated secondary antibodies, were utilized for detection. The blots were visualized using the enhanced chemiluminescence (ECL) HRP substrate (Millipore) and analyzed with a ChemiDoc Imager (Bio‐Rad). Protein expression levels were normalized to GAPDH.

### 2.12 Statistical Analyses

Statistical analyses were performed using R (version 4.2.2). For comparisons between two groups, Student's *t*‐test was applied only after verifying the underlying assumptions. Specifically, the normality of data distribution was assessed using the Shapiro–Wilk test, and homogeneity of variances was evaluated using Levene's test. In cases where the normality assumption was satisfied (*p* > 0.05) but variances were unequal (*p* < 0.05), Welch's *t*‐test was employed. If the normality assumption was violated, the non‐parametric Mann–Whitney U test was used instead. All tests were two‐sided, and a *p*‐value < 0.05 was considered statistically significant. These statistical details are explicitly reported in the corresponding Results section.

## 3 Results

**FIGURE 1:**
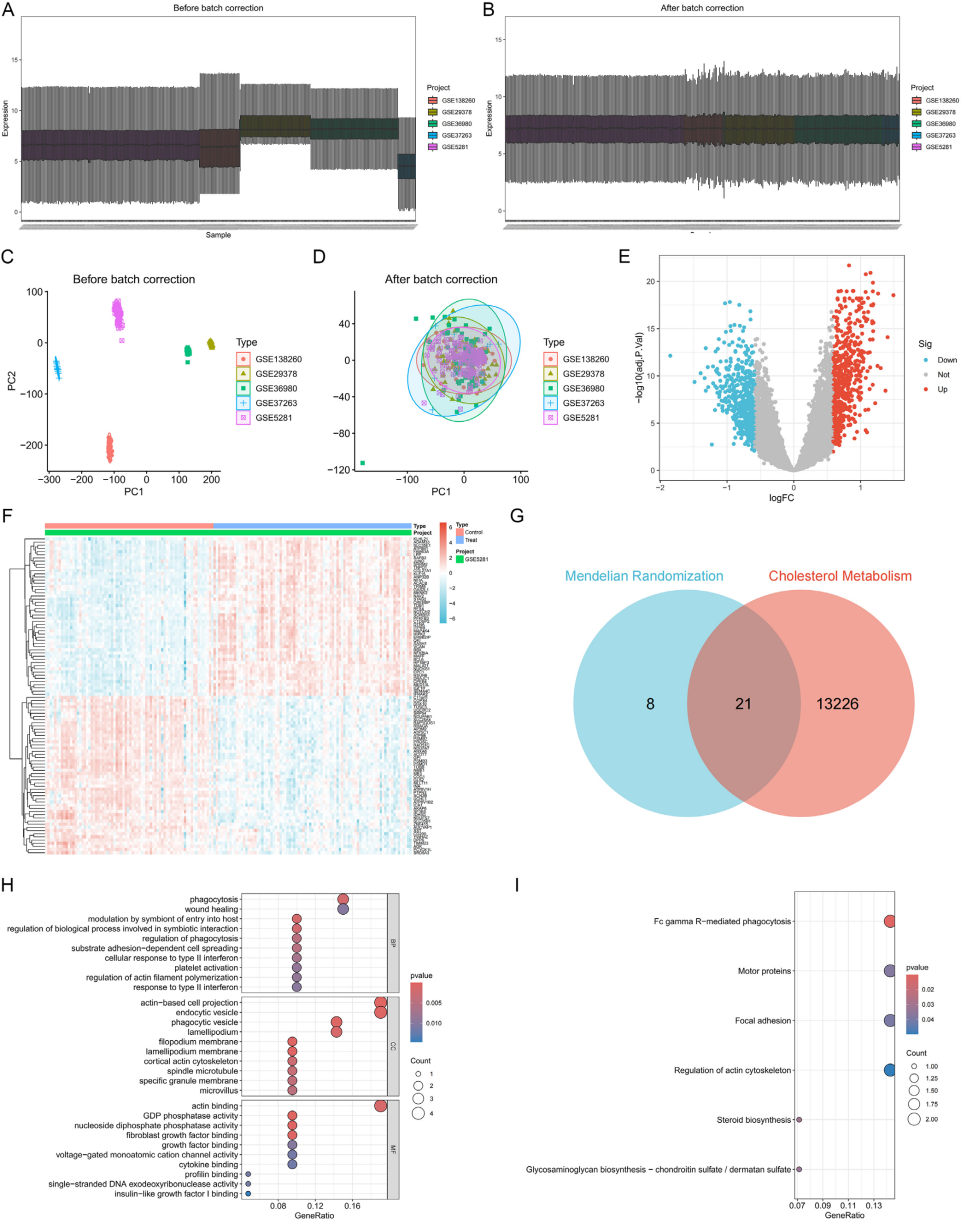
Comprehensive gene expression analysis. (A–D) Sample normalization process. (E) Volcano plots of differentially expressed genes (DEGs). (F) Heatmap of the top 50 genes. (G) Intersection of genes selected by Mendelian randomization and related to cholesterol metabolism. (H) GO enrichment analysis for hub genes. Presentation of the top 10 results for BP, CC, and MF. (I) KEGG enrichment analysis for hub genes.

In this study, gene expression raw data and platform information were downloaded from the GEO database. Subsequently, the data were reannotated and normalized (Figure 1A–D). ComBat and limma were performed to remove the batch effects and calculated corresponding kBET rejection rates to compare the 2 methods; the results indicated ComBat was the better method to remove batch effects (Figure S1 & Table S2). The volcano plot (Figure 1E) shows the differentially expressed genes (DEGs) between Alzheimer's disease and non‐demented controls, and the expression heatmap (Figure 1F) illustrates the top 50 genes identified by differential expression analysis. Then, we used Mendelian randomization (MR) to identify 29 genes causally associated with Alzheimer's disease from the DEGs. The identified genes were intersected with genes related to cholesterol metabolism, resulting in 21 hub genes (Figure 1G). GO (Figure 1H) and KEGG (Figure 1I) enrichment analyses were performed on the 21 hub genes to explore their biological functions and pathways. All DEGs between normal and AD samples with the criterion of |log_2_FC| > 0.585 are shown in Table S3.

**FIGURE 2:**
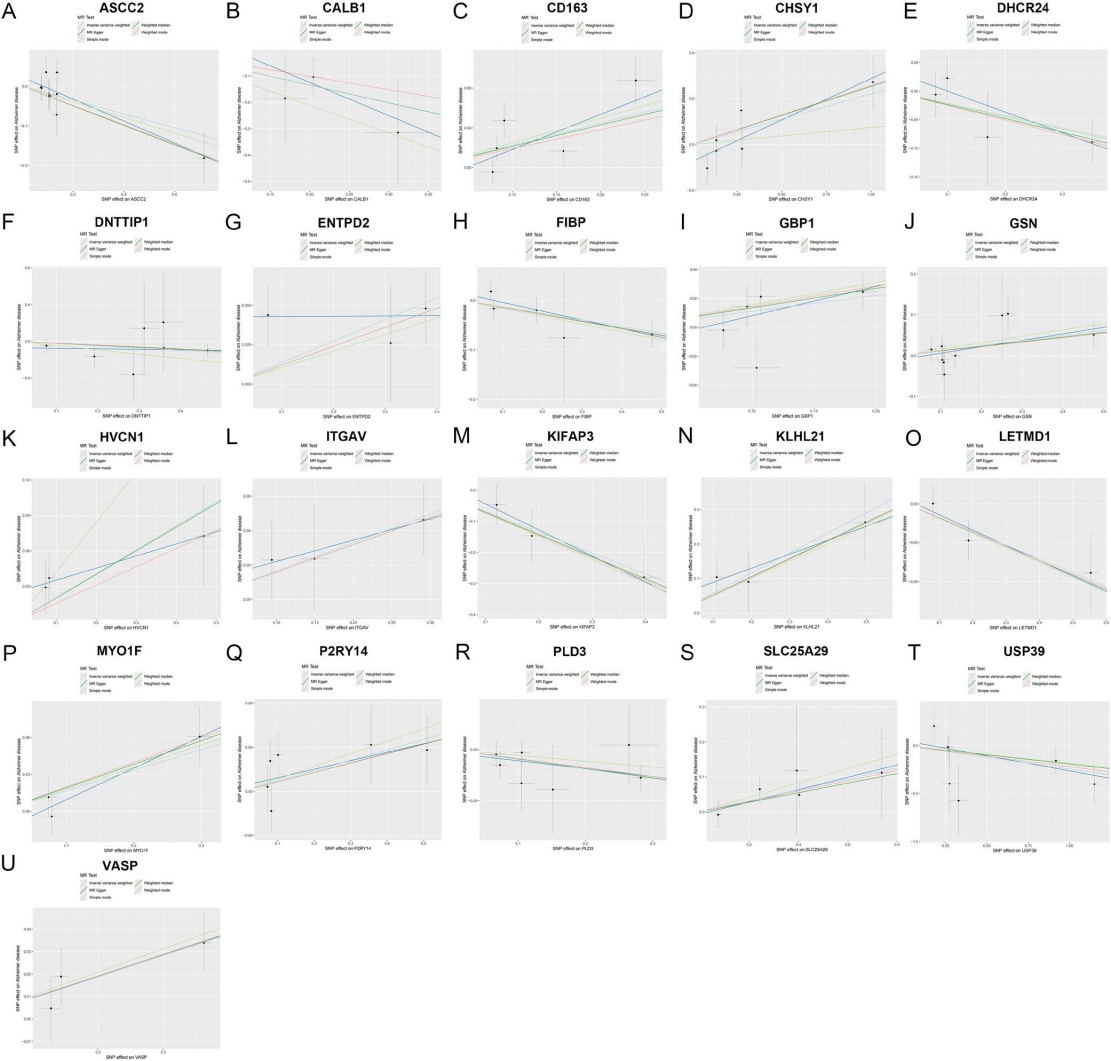
Scatter plot of MR analysis between 21 hub genes—ASCC2 (A), CALB1 (B), CD163 (C), CHSY1 (D), DHCR24 (E), DNTTIP1 (F), ENTPD2 (G), FIBP (H), GBP1 (I), GSN (J), HVCN1 (K), ITGAV (L), KIFAP3 (M), KLHL21 (N), LETMD1 (O), MYO1F (P), P2YR14 (Q), PLD3 (R), SLC25A29 (S), USP39 (T), and VASP (U) and Alzheimer's disease.

The scatter plots of the causal effect estimates between 21 hub genes and AD using different MR methods are shown in Figure 2. Additionally, Figure S2 presents a forest plot illustrating the results of the MR analysis for each SNP utilizing the Inverse Variance Weighted (IVW) model. Detailed results of validation analyses are provided in Table S4.

**FIGURE 3:**
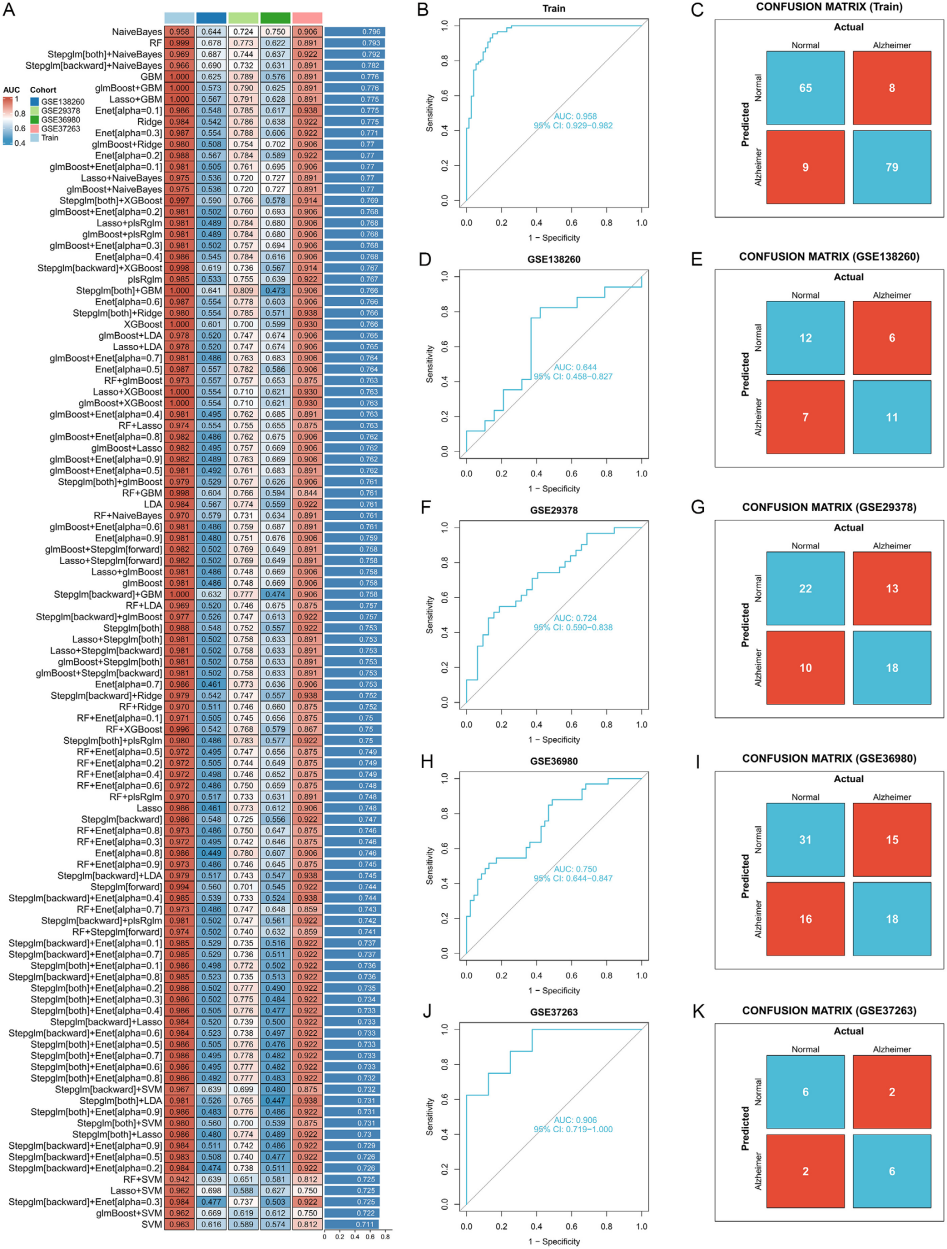
Construction of the predictive model and selection of prognostic genes. (A) AUC values of 113 machine learning algorithm combinations across four testing cohorts. (B) ROC curve of the models in the training dataset (GSE5281). (C) Confusion matrices for the models of the training dataset (GSE5281). (D–K) ROC curves and confusion matrices of the models in each dataset.

**FIGURE 4:**
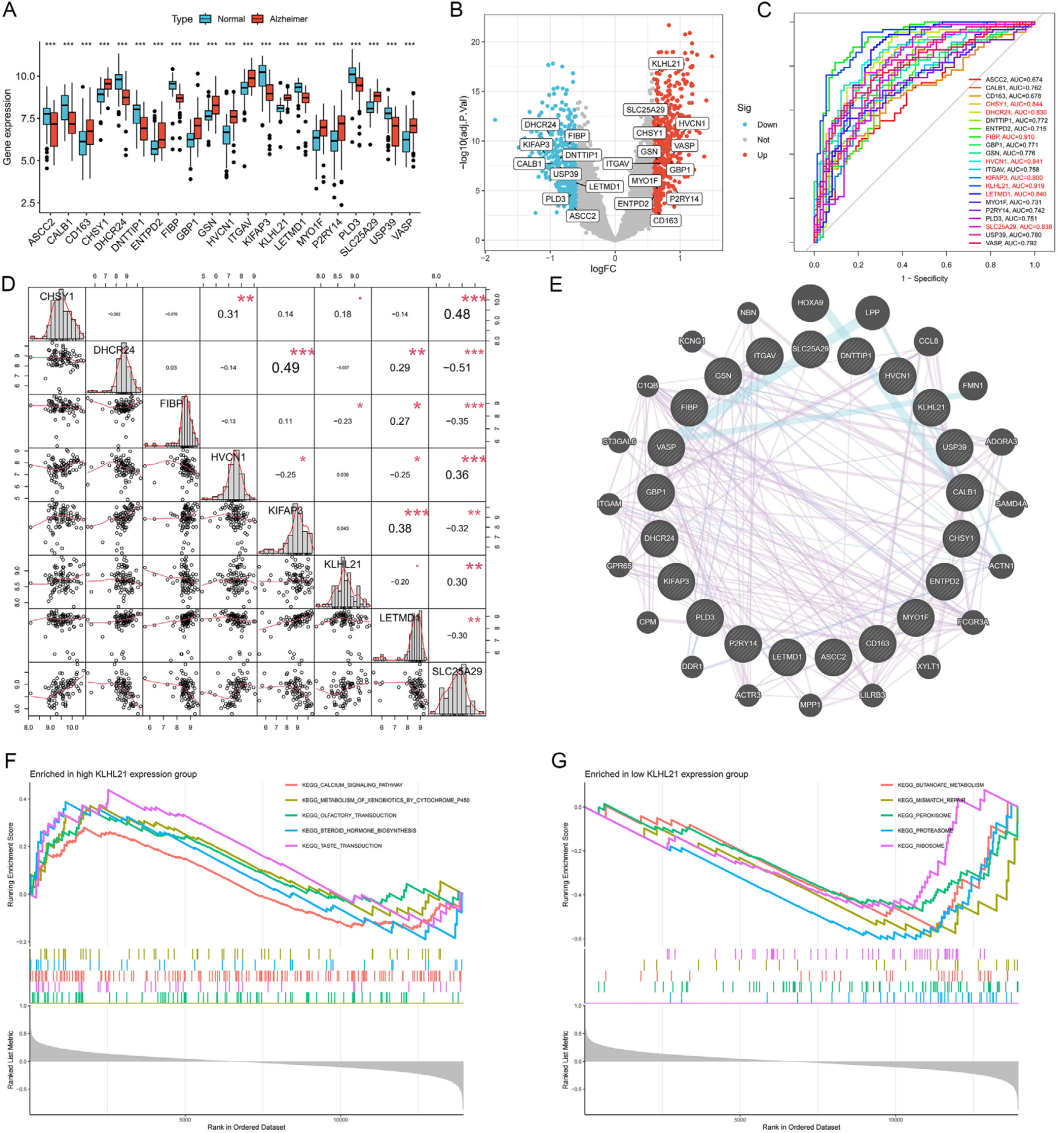
(A) The Expression of hub genes in Alzheimer's and normal groups. (B) Volcano plots depicting hub genes in Alzheimer's and normal groups. (C) ROC curves for hub genes in the Naïve Bayes model. (D) Heat map of the correlation between eight screened genes. (E) The Protein–Protein Interaction (PPI) Network of the screened genes. (F) GSEA analysis of functions and pathways based on high expression of KLHL21. (G) GSEA analysis of functions and pathways based on the low expression of KLHL21.

To identify the most robust diagnostic model (Figure 3A), we applied 12 machine learning algorithms combined with a 10‐fold cross‐validation using 21 hub genes. Specifically, this analysis was performed in the training dataset (GSE5281) and 4 external validating datasets (Figure 3B–K). The final model with the best performance was developed using the Naive Bayes algorithm. The differences in the expression of these 21 hub genes between Alzheimer's disease patients and healthy controls are shown in Figure 4A,B. Next, we evaluated the diagnostic significance of these 21 hub genes with ROC curves; their AUC exceeded 0.8. As shown in Figure 4C, using the Naive Bayes algorithm, 8 key genes were identified: CHSY1, FIBP, DHCR24, HVCN1, KIFAP3, KLHL21, LETMD1, and SLC25A29. In the correlation matrix heat map (Figure 4D), KLHL21 and FIBP showed varying degrees of correlation with other genes. The STRING tool was used to acquire protein–protein interactions (PPIs) for the 8 key genes in the network (Figure 4E). Gene set enrichment analysis (GSEA) revealed that KLHL21 overexpression is involved in regulating steroid hormone biosynthesis (Figure 4F), while low expression of KLHL21 is associated with butanoate metabolism (Figure 4G).

**FIGURE 5:**
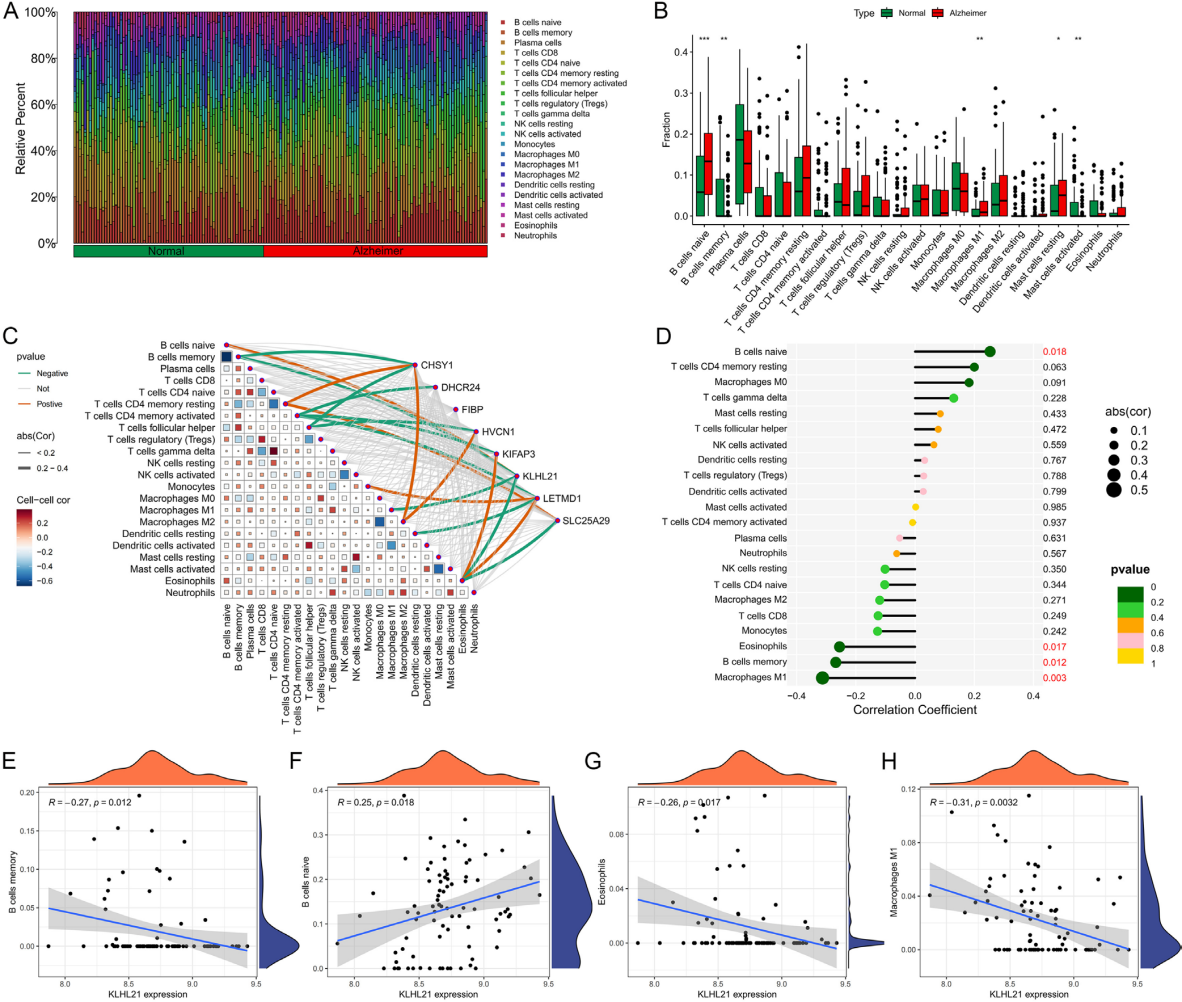
(A) Relative proportion of immune cell infiltrates in Alzheimer's and normal groups. (B) Comparison of 22 immune cell types in Alzheimer's and normal groups. Green indicates normal, and red indicates Alzheimer's groups. (C) Heatmap showing the regulatory relationships between immune cells and screened genes. (D) Bar charts illustrating the regulatory relationships between KLHL21 and immune cells. (E–H) Correlation analysis of KLHL21 and memory B cells (E), naïve B cells (F), Eosinophils (G), and Macrophages M1 (H).

We used CIBERSORT to predict immune cell proportions and examine infiltration patterns in the AD and control groups (Figure 5A,B). The results revealed high infiltration of naive B cells, resting mast cells, and M1 macrophages in the AD group, while activated mast cells and memory B cells were significantly infiltrated in the control group. Correlation network analysis revealed complex relationships between immune cell types and gene expression levels (Figure 5C). KLHL21 showed a significant association with various immune cells, suggesting that KLHL21 may play a role in immune response regulation. Detailed correlation analysis showed that KLHL21 expression was significantly positively correlated with naive B cells (*p* = 0.018) and negatively correlated with M1 macrophages (*p* = 0.003), memory B cells (*p* = 0.012), and eosinophils (*p* = 0.017) (Figure 5D–H). Moreover, 4 additional methods were used to evaluate the differences in immune cell infiltration in normal and AD samples (Figure S3).

**FIGURE 6:**
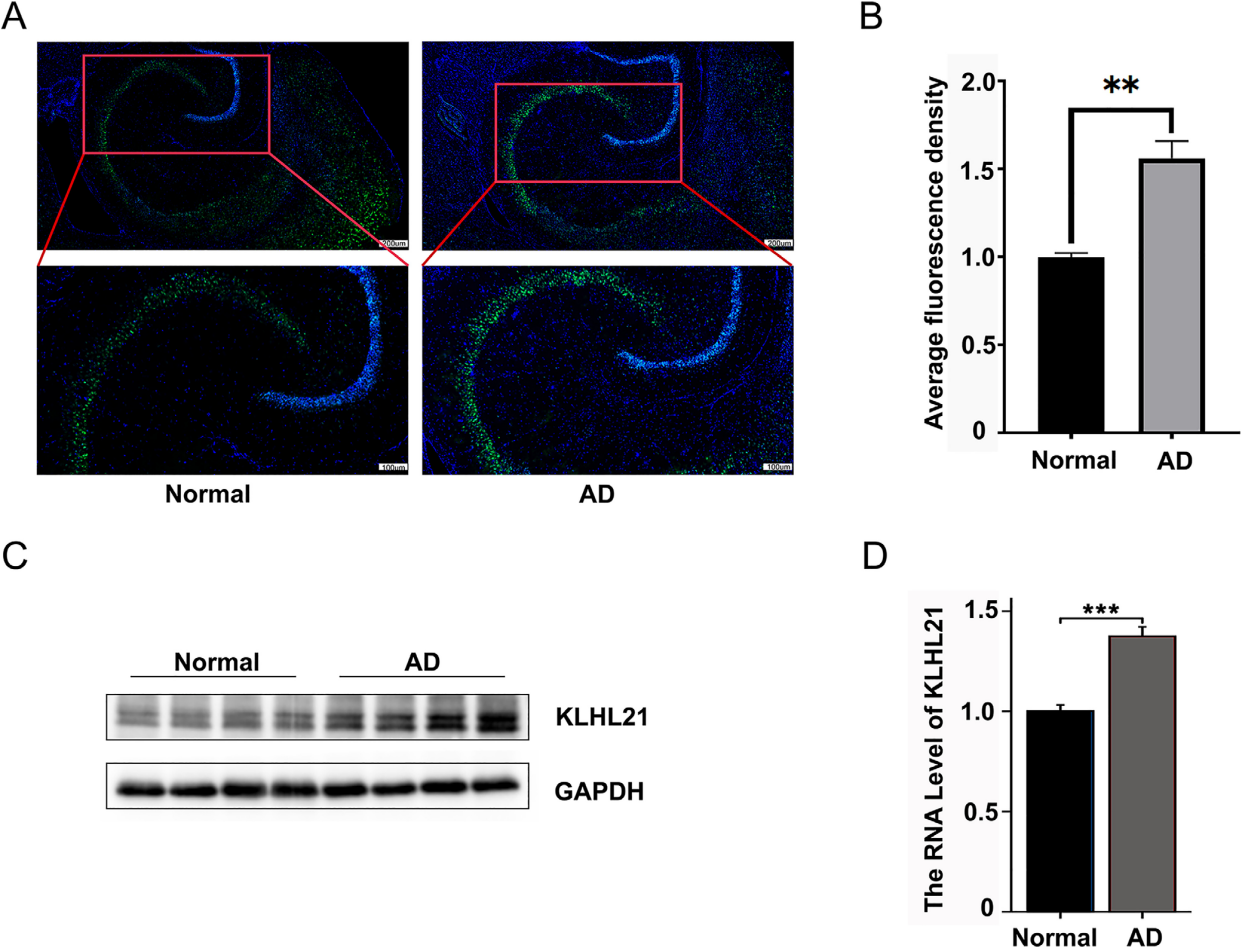
(A) Immunofluorescence staining of KLHL21 in brain tissues from Alzheimer's disease mice and controls. Blue, nuclei; Green, KLHL21. Scale bar = 200 μm and 100 μm. (B) Histogram of relative protein expression in brain tissues from Alzheimer's disease mice and controls. (C) Western blot images. (D) qPCR analysis. Data are presented as means ± SD. Student's *t*‐test, **p < 0.01.

To validate these findings, we used APP23 mice, a widely utilized Alzheimer's disease mouse model that overexpresses the human APP gene with the Swedish double mutation and that begins to show spatial memory deficits at 3 months of age [17]. Immunofluorescence staining was performed on the other half of the brain tissues from APP23 and wild‐type mice. The results showed that KLHL21 expression was higher in Alzheimer's disease mice (Figure 6A,B). This was further confirmed by Western blot (Figure 6C) and qPCR (Figure 6D).

## 4 Disscusion

AD is a prevalent neurodegenerative disorder characterized by progressive cognitive decline, particularly affecting memory and executive functions. This condition poses significant challenges not only to affected individuals but also to their families and health systems globally. The World Health Organization has indicated that the prevalence of Alzheimer's disease is expected to increase sharply [3]. This highlights the urgent need to advance therapeutic approaches and early detection methods to reduce the socio‐economic impact of the disease. Despite various pharmacological interventions, effective treatment options remain limited. This highlights the urgent need for novel biomarkers and therapeutic targets to aid in early detection and therapeutic interventions [18]. This study focuses on the transcriptomic characteristics of AD, specifically investigating genes associated with cholesterol metabolism, which previous research has linked to the pathophysiology of AD [19]. The discussion delves into the implications of these findings. It includes the potential for these genes to serve as biomarkers for early diagnosis and as targets for therapeutic interventions, thereby providing insights into the underlying mechanisms of Alzheimer's disease progression.

To connect this focus with our gene expression analysis, the identification of differentially expressed genes (DEGs) between AD patients and control groups is crucial for elucidating the underlying mechanisms of the disease. In our analysis of the transcriptomic datasets from AD patients, we identified multiple DEGs that exhibited significant expression differences when compared to control groups. The biological significance of these genes is important to consider, as they may play critical roles in the pathophysiology of AD. For example, genes involved in synaptic function, inflammation, and neurodegeneration were among those identified, aligning with previous findings in the literature that suggest dysregulation of these pathways contributes to cognitive decline in AD patients [20, 21]. Furthermore, refining the criteria and methods used for DEG selection is essential to enhance the specificity and sensitivity of our findings. Employing advanced statistical techniques, such as machine learning algorithms and Mendelian randomization analysis—a method that uses genetic variants to infer causal relationships—may improve the accuracy of DEG detection and provide a more comprehensive understanding of their biological functions within the context of AD [22, 23, 24].

The causal relationships established through Mendelian randomization analysis are pivotal in elucidating the genetic architecture of AD [25, 26]. We identified 29 genes that have confirmed causal links to the disease, expanding the knowledge of genetic predisposition to AD. Understanding these causal relationships not only provides insights into the mechanisms driving disease progression but also highlights potential therapeutic targets. It is important to consider how these causal genes can be utilized in drug development strategies. For instance, if certain genes are implicated in lipid metabolism disturbances, as seen with the APOE gene's association with cholesterol metabolism, this could inform the design of interventions aimed at restoring metabolic balance in AD patients [11]. Future studies should validate these causal relationships using functional assays and examine their clinical implications.

The intersection of the identified causal genes with cholesterol metabolism presented a compelling avenue for further exploration. In our investigation into causal relationships, Mendelian randomization analysis revealed 29 genes with a direct causal relationship to AD; 21 of these intersected with cholesterol metabolism‐related genes. This observation suggests that dysregulation of cholesterol metabolism may significantly influence the progression of AD [14, 27]. Prior research has established that altered cholesterol levels can influence amyloid precursor protein processing and tau phosphorylation—both critical elements in AD pathology [28, 29, 30]. Therefore, investigating the biological mechanisms connecting these genes to cholesterol metabolism could illuminate novel therapeutic strategies. For instance, pharmacological agents or lifestyle interventions targeting cholesterol levels might mitigate or even prevent the progression of AD by addressing underlying metabolic dysregulation [31, 32]. Future research should aim to characterize these pathways further and assess how they might be modulated to produce beneficial effects in AD patients.

The construction of diagnostic models based on machine learning techniques, such as LASSO and random forests, underscores the potential for these methodologies to revolutionize early diagnosis in AD. By identifying a panel of eight genes with high diagnostic accuracy, our model has the potential to facilitate timely interventions that could alter the disease trajectory. The model's performance, confirmed by rigorous cross‐validation, suggests it could be a reliable tool for early diagnosis, essential for timely intervention in AD management [33]. One hundred thirteen algorithm combinations were combined to fit the prediction model. The advantage of the integrative procedure is to identify the signature with a consistent predictive performance based on multiple machine learning methods and their combinations. Algorithm combinations can further reduce the dimension of variables and make the model simpler and more translatable. So, instead of messing around with these machine learning algorithms and abusing them, we paired them up and validated the results to find the best combination of algorithms. Future research should aim to refine this model further by incorporating larger datasets and exploring additional machine learning methodologies to enhance predictive power and clinical applicability. The integration of these predictive models into clinical practice could revolutionize diagnostic standards for individuals at risk of AD, paving the way for personalized therapeutic strategies.

The observed differences in immune cell compositions among the study cohorts highlight the intricate relationship between the immune system and AD pathology. Our findings indicate significant variations in immune cell types, which may reflect the neuroinflammatory processes that are increasingly recognized as contributors to AD. Understanding how these immune cells interact with neurodegenerative pathways could open new therapeutic avenues. This is particularly relevant in the context of immunotherapy strategies [34, 35]. There is a growing body of literature supporting the role of immune modulation in AD, suggesting that targeting specific immune pathways may ameliorate disease symptoms or slow progression [34, 36]. Future investigations should focus on elucidating the functional roles of these specific immune cell populations in the context of AD and how they might be leveraged for therapeutic interventions. Our study utilized CIBERSORT to computationally infer immune cell abundances from bulk RNA‐seq data, revealing a potentially significant association between KLHL21 expression and specific immune cell subsets. We acknowledge, however, that deconvolution approaches applied to bulk transcriptomes involve inherent limitations, as they rely on algorithmic estimation rather than direct measurement, and different methods may yield varying results. To assess the robustness of our observations, we employed four additional deconvolution algorithms, which returned generally consistent patterns of immune cell infiltration associated with KLHL21 expression. Nevertheless, these findings remain computational inferences. It suggests a plausible role of KLHL21 in the neuroimmune landscape of AD; future studies using direct measurement techniques such as flow cytometry or immunohistochemistry on well‐characterized cohorts are necessary to confirm these associations. Thus, the current results highlight an interesting hypothesis‐generating link rather than a definitive biological mechanism.

Machine learning identified eight key genes associated with AD. Among these, Kelch‐like family member 21 (KLHL21) exhibited superior predictive ability in diagnostic modeling, achieving an AUC of 0.919. The Gene Set Enrichment Analysis (GSEA) revealed that KLHL21 is enriched in pathways related to steroid hormone biosynthesis, further confirming the close relationship between cholesterol metabolism and AD. KLHL21, known for its association with the malignant behavior of various tumors [37, 38, 39], has received limited attention in the context of AD, and its relevance to tumor biology warrants further investigation regarding its role in AD. Immunofluorescence staining in the AD mouse model corroborated the increased expression of KLHL21 (*p* < 0.01), reinforcing its potential as a biomarker for the disease. The experimental validation enhances the credibility of our findings and supports the hypothesis that KLHL21 may play a pivotal role in the pathogenesis of AD. Nevertheless, it is essential to consider the limitations of the current study. These include the relatively small sample size and the need for multi‐center validation to establish the generalizability of our findings. Moving forward, it will be crucial to explore the therapeutic implications of KLHL21, investigate its potential role in modulating disease progression, and examine its interactions with established therapeutic modalities.

This study has several limitations that should be taken into account. First, because we relied on transcriptomic data, we could not validate our findings with wet laboratory experiments. Such experiments are essential to confirm the biological significance of the differentially expressed genes we identified. Additionally, the sample size in this study may limit the generalizability of our conclusions. A larger cohort, which could yield more reliable insights, is needed. Moreover, the potential for batch effects arising from the variety of datasets employed may not have been completely addressed, which could affect the reliability of our results. These issues highlight the necessity for further research that includes a wider range of data types and larger sample sizes. Such studies are essential to validate our findings and clarify the complex mechanisms involved in Alzheimer's Disease (AD).

## 5 Conclusions

In conclusion, our research identified various genes linked to AD and developed a diagnostic model with promising clinical potential. By analyzing immune cell composition and metabolic pathways, we provided valuable insights into the disease mechanisms. While our findings lay a crucial foundation for future research, further studies are necessary to validate these results and deepen our understanding of AD's complex biology. This will ultimately support the development of effective therapeutic strategies.

## Author Contributions

Hongrui Zhu, Sheng Wang, and Keqiang He planned and oversaw the investigation. Rui Sun and Xu Wang conducted the experiments, drafted the manuscript, and formatted it. The figures were designed by Zaibao Wang, Chunliu Li, Qing Shao, and Xiangru Liu. Furthermore, each author offered insightful criticism and contributed to the manuscript's development.

## Funding

This work was supported by Hubei Chen Xiaoping Science and Technology Development Foundation (CXPJJH125001‐2537) and the Health Research Project of the Anhui Provincial Health Commission (AHWJ2023BAc20089).

## Ethics Statement

All experimental procedures carried out in this research adhered to the protocols established by the Committee for the Care and Use of Laboratory Animals at the University of Science and Technology of China. Additionally, the study was authorized by the Laboratory Animal Center of the First Affiliated Hospital of the University of Science and Technology of China, Anhui Provincial Hospital, and received ethical approval under the number 2022‐N(A)‐175.

## Consent

The authors have nothing to report.

## Conflicts of Interest

The authors declare no conflicts of interest.

## Supporting information


**Figure S1:** ComBat and limma were performed to remove the batch effects and calculated corresponding kBET rejection rates to compare the 2 methods; the results indicated that ComBat was the better method to remove batch effects.

**Figure S2:** Leave‐one‐out sensitivity analysis of 21 genes.

**Figure S3:** Four additional methods were used to evaluate the differences in immune cell infiltration in normal and AD samples.

**Table S1:** All enriched pathways and relative statistical results of the GSEA analyses.

**Table S2:** kBET rejection rates of ComBat and limma methods for removing the batch effects.

**Table S3:** All DEGs between normal and AD samples with the criterion of |log_2_FC| > 0.585.

**Table S4:** Supplemental statistical validation of MR analyses.

## Data Availability

The data that support the findings of this study are openly available in sun00814/ad260118 at https://doi.org/10.5281/zenodo.18289856.
