# Supplementary figures and images for: Transcriptomic Insights Into Alzheimer's Disease: Differentially Expressed Genes and Cholesterol Metabolism
Source: CNS Neurosci Ther. 2026 Mar 19;32(3):e70833. doi: 10.1002/cns.70833 (PMC13093267; doi:10.1002/cns.70833)

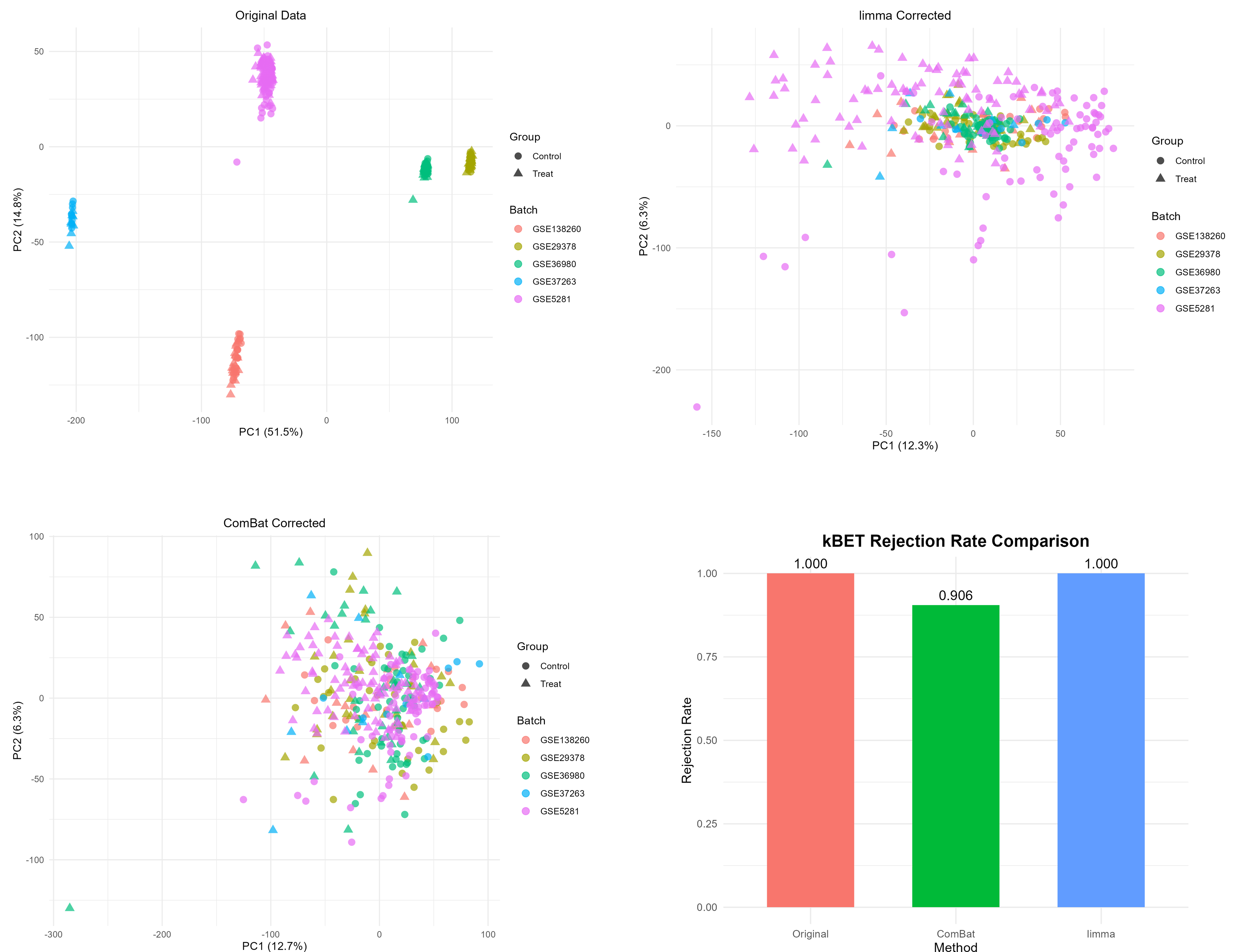

Supplement: Supplementary file 1 — Figure S1: ComBat and limma were performed to remove the batch effects and calculated corresponding kBET rejection rates to compare the 2 methods; the results indicated that ComBat was the better method to remove batch effects. [file CNS-32-e70833-s003.tif]

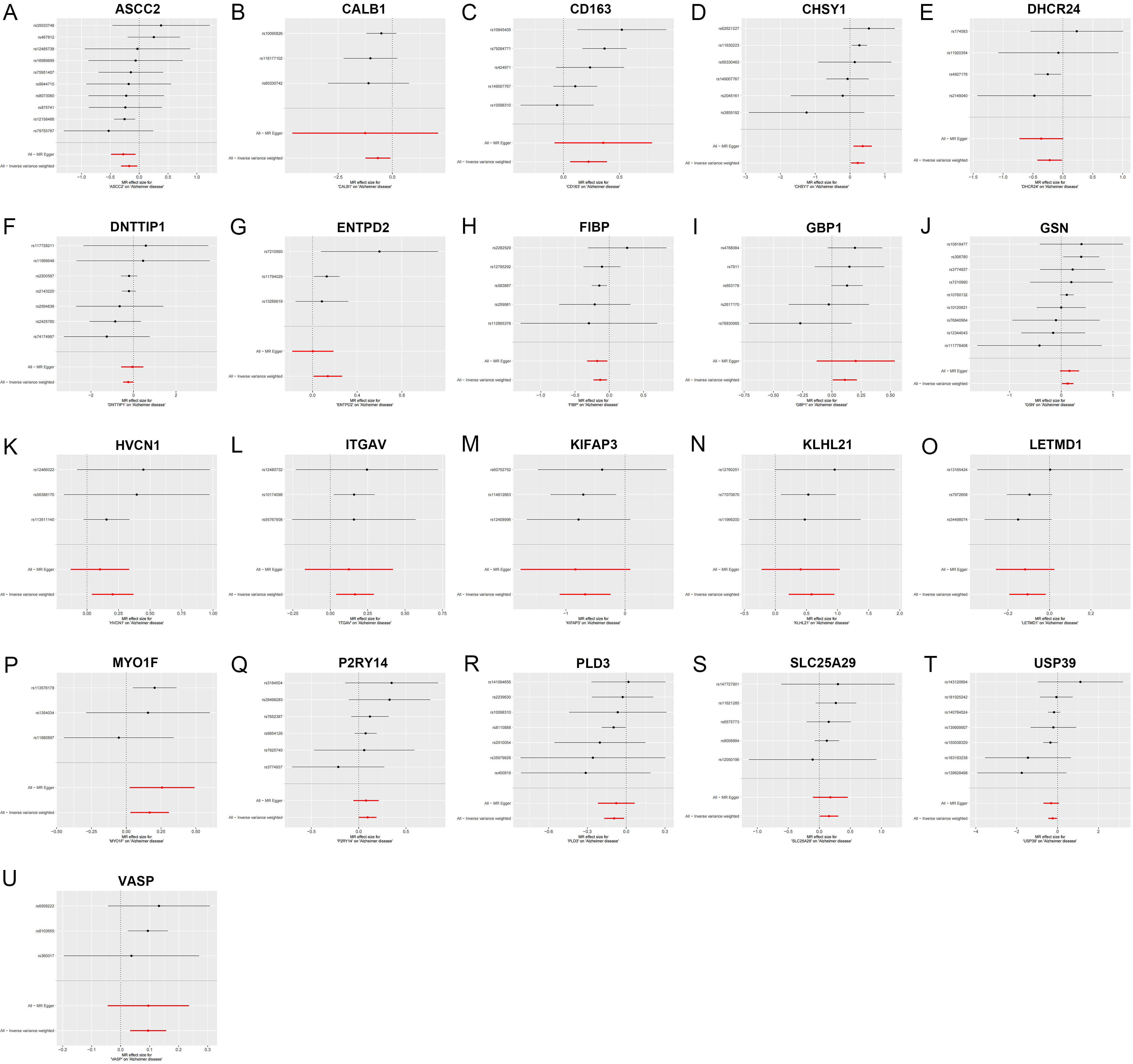

Supplement: Supplementary file 2 — Figure S2: Leave‐one‐out sensitivity analysis of 21 genes. [file CNS-32-e70833-s007.tif]

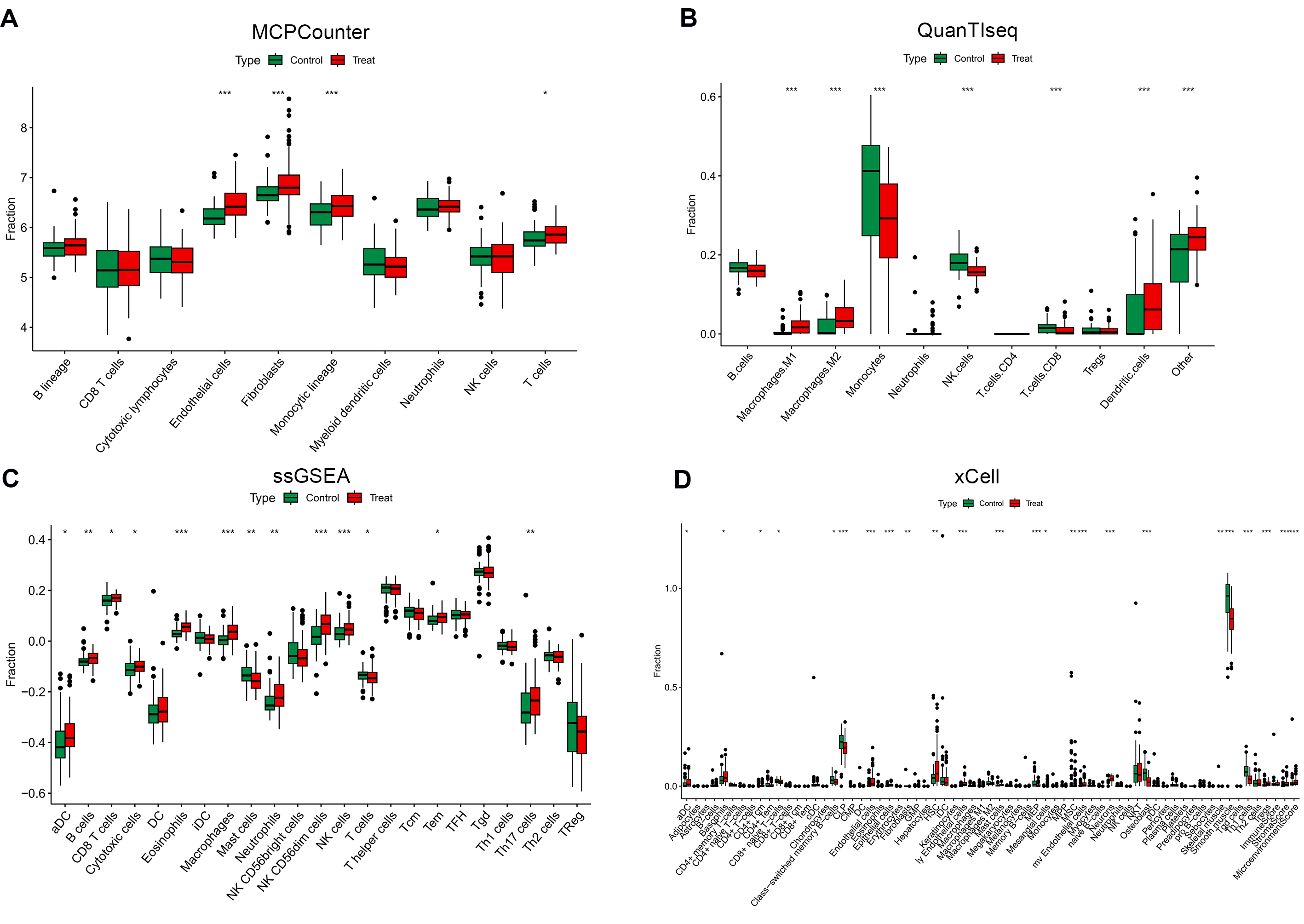

Supplement: Supplementary file 3 — Figure S3: Four additional methods were used to evaluate the differences in immune cell infiltration in normal and AD samples. [file CNS-32-e70833-s001.tif]
